# Coaching Community Health Volunteers in Integrated Community Case Management Improves the Care of Sick Children Under-5: Experience from Bondo, Kenya

**DOI:** 10.5334/ijic.3971

**Published:** 2018-10-24

**Authors:** Makeba Shiroya-Wandabwa, Mark Kabue, Dyness Kasungami, Jonesmus Wambua, Dan Otieno, Charles Waka, Augustine Ngindu, Christine Ayuyo, Sanyu Kigondu, Julius Oliech, Isaac Malonza

**Affiliations:** 1 Independent Consultant, Nairobi, KE; 2 Jhpiego Baltimore, Baltimore, US; 3 John Snow, Inc., Boston, US; 4 Jhpiego Kenya, Nairobi, KE; 5 Independent Consultant, Kisumu, KE; 6 Ministry of Health, Nairobi, KE

**Keywords:** child health, training, community, hard-to-reach, task shifting

## Abstract

**Background:**

Shortages of healthcare workers is detrimental to the health of communities, especially children. This paper describes the process of capacity building Community Health Volunteers (CHVs) to deliver integrated preventive and curative package of care of services to manage common childhood illness in hard-to-reach communities in Bondo Subcounty, Kenya.

**Methods:**

A pre-test/post-test single-group design was used to assess changes in knowledge and skills related to integrated community case management (iCCM) among 58 Community Health Volunteers who received a six-day iCCM clinical training and an additional 3-week clinical coaching at health facilities. Thereafter, community health extension workers and health managers provided supportive supervision over a six-month period. Skills were assessed before the six-day training, during coaching, and after six months of iCCM implementation.

**Results:**

CHVs knowledge assessment scores improved from 54.5% to 72.9% after the six-day training (*p* < 0.001). All 58 CHVs could assess and classify fever and diarrhoea correctly after 3–6 weeks of facility-based clinical coaching; 97% could correctly identify malnutrition and 80%, suspected pneumonia. The majority correctly performed four of the six steps in malaria rapid diagnostic testing. However, only 58% could draw blood correctly and 67% dispose of waste correctly after the testing. The proportion of CHV exhibiting appropriate skills to examine for signs of illness improved from 4% at baseline to 74% after 6 months of iCCM implementation, *p* < 0.05. The proportion of caregivers in intervention community units who first sought treatment from a CHV increased from 2 to 31 percent (*p* < 0.001).

**Conclusions:**

Training and clinical coaching built CHV’s skills to manage common childhood illnesses. The CHVs demonstrated ability to follow the Kenya iCCM algorithm for decision-making on whether to treat or refer a sick child. The communities’ confidence in CHVs’ ability to deliver integrated case management resulted in modification of care-seeking behaviour.

## Introduction

### Problem Statement

In Kenya, as in most countries in sub-Saharan Africa, the primary causes of preventable death in children after the newborn period are diarrhoea, malaria, and pneumonia, with malnutrition as an underlying cause of all three [1]. Kenya’s Under-5 Mortality Rate fell by about 50% from approximately 99 per 1,000 live births in 2003 to 52 per 1,000 live births in 2014 [2] The decline was due to the World Health Organization Expanded Programme on Immunization (EPI) against six diseases: tuberculosis, measles, diphtheria, whooping cough, tetanus and polio as well as the low cost high impact interventions to control diarrhoea, such as oral rehydration therapy (ORT). Kenya is experiencing low health worker retention and health worker shortages of all cadres but particularly specialized health professionals and low retention, especially in hard-to-reach areas (distance > 10 km from community unit to link health facility). The 2008 Kenya Ministry of Health National human resources for health strategic plan indicated that Kenya had a 29% vacancy level [3,4] There is an average 1.5 health workers per 1,000 people in Kenya, which is below the minimum staffing threshold of 2.3 per 1,000 recommended by the World Health Organization (WHO). There are even fewer health workers in hard-to-reach and marginalized regions of the country [5] Health worker shortages result in reduced coverage of high-impact life-saving interventions. Other barriers to timely access to healthcare include long distance to health facilities (defined as over 5 kilometres in Kenya), limited caregiver health knowledge, and sociocultural isolation [6,7,8,9]. In response to the urgent need to address health worker shortages and increase access to life-saving treatments, many Ministries of Health (MoH) in developing countries are engaging community health workers (CHWs) to increase coverage of health services, according to the WHO [10].

### Background

Community-based service providers are trusted community members chosen by the community because of their integrity and commitment to the health and wellbeing of the community. They are trained to treat and advise about the health problems of individuals and the community, and working in close relationship with health service providers. Community health volunteers —if appropriately trained, supervised, and supported with an uninterrupted supply of essential medications and supplies—can identify and correctly treat most children who have common childhood diseases that contribute significantly to childhood morbidity and mortality [10,11]. Evidence from Malawi shows that 68% of classifications of common illnesses by a cadre of workers called “health surveillance assistants” agreed with assessments done by physicians, and 63% of children were prescribed appropriate medication [12]. The health surveillance assistants are paid by the MoH. The issue of remuneration has plagued the deployment of these service providers in many countries. In Kenya, the terms “workers” has been replaced with “volunteers” in line with the country’s labour laws, thus they are referred to as community health volunteers (CHVs). The (iCCM)/UNICEF integrated community case management strategy integrates a preventive and curative package of health services to improve health outcomes of sick children under-5 by equipping CHVs with the necessary skills to manage children with diarrhoea, suspected pneumonia, and malaria in order to increase coverage of health services for under-5s [13,14,15,16]. In Nepal, which has more than 20 years of experience in community-based management of child illness, 69% of the under-5 population has access to treatment. Across the country, both the case fatality rate for acute diarrhoea and the proportion of severe pneumonia among acute respiratory infection cases have decreased significantly [17]. In Ghana, 92% of caregivers of sick children sought treatment from community-based service providers trained to manage pneumonia and malaria; most caregivers sought care for their children within 24 hours of onset of fever [18]. Additional evidence from a cluster randomized trial in Zambia showed that 68% of children with pneumonia received early and appropriate treatment from CHVs and that overtreatment of malaria significantly declined over a 12-month period [19]. In Ethiopia, a cadre of community case management workers known as “health extension workers,” who are deployed in remote communities, delivered two and a half times as many treatments for the three major childhood infectious illness diseases as all the facility-based providers in the same district [20]. In all these countries, community case management is considered a major contributing factor to increasing access to timely case management and decreasing preventable deaths.

In January 2015, the Kenyan Ministry of Health launched the national iCCM implementation framework and action plan, which is a platform for community management of childhood diarrhoea, malaria, pneumonia, neonatal illness, and malnutrition [21]. The framework is anchored in the community health strategy [22] and the *Child Survival and Development Strategy* [23], which address key areas including policy, management of cases and the commodity supply chain, and supervision. In Kenya, CHVs are trained to treat under-5 children with diarrhoea using oral rehydration salts (ORS) and zinc, to diagnose malaria with a malaria rapid diagnostic test (mRDT) and treat it with artemisinin combination therapy, and to refer suspected pneumonia, mild to moderate malnutrition, and sick newborn to a health facility [21]. Before being certified as CHVs, all candidates receive basic training which focuses on health and development, health promotion, and the Kenya community-based essential health package [22]. To provide integrated Community Case Management, Community Health Volunteers take an additional six-day training course in case management of fever, diarrhoea, and cough/fast breathing.

At the time of this study, every CHV in the 26 community units (CUs) of Bondo Subcounty received a monthly allowance of KES 2,000 KES (about USD 23). The payment of this allowance started about 5 years before this study and was continued throughout the 18-month duration of the study. Before iCCM was introduced in the CHVs in all the Community Units routinely spent 1–2 days at health facilities assisting in tasks such as giving health talks to clients and scheduling appointments.

Building a competent and sustainable community-level health workforce as service providers of iCCM is a challenge. While the use of CHVs to deliver iCCM promises to increase timely access to life-saving treatments, scaling up this approach must also ensure that the CHVs who are assessing and treating sick children are properly trained and supervised. Structured CHV training that ensures the development of skills may serve as a key incentive for both attracting and retaining CHVs [24]. The goal of the training is to build the skills of CHVs; the most effective type of training seems to be a combination of didactic training with interactive sessions, practicums, and fieldwork [14]. In rural Uganda, CHVs trained on use of respiratory timers for diagnosing pneumonia and malaria rapid diagnostic tests were able to adequately perform the following skills when managing children under 5: 97% taking history, 96% using timers, 96% using malaria rapid diagnostic tests, and 85% breath reading [16].

A key element in developing a competent CHV workforce is mentorship that includes coaching in a health facility by a nurse or clinical officer to help the CHVs acquire the relevant clinical skills to properly manage childhood diseases. Another study in Uganda reported that CHV mentorship resulted in good performance in malaria and pneumonia knowledge (72%), and capacity to elicit signs and symptoms by 50% of the CHVs [15].

This paper describes the process of equipping the CHVs to provide iCCM services, and reports on the level of CHVs’ knowledge and skills after six months of implementing iCCM in the community. The data analysed were part of an iCCM implementation research study in Bondo Subcounty, Kenya, that was designed to assess whether the addition of the iCCM technical module onto the existing Kenyan community health platform [22] improves coverage and quality of services addressing childhood illnesses at community and facility level. Bondo was selected for this study because at the time of the study, it had one of the highest infant mortality rates in Kenya, at 110 infants per 1,000 live births, and an under-five mortality rate of 208 per 1,000 live births—four times the national average [25].

### Theory of Change

**Figure 1:**
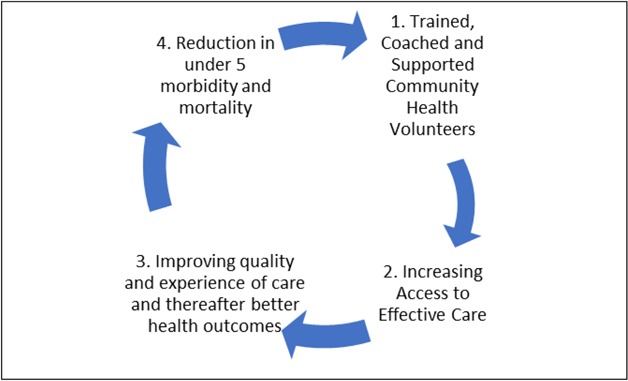
Theory of change – iCCM contribution to reduction of under 5 morbidity and mortality.

A theory of change was developed by the investigators that shows the results pathway starting with the intervention and ending with the goal of reducing mortality (See Figure 1).

## Methods

### Setting

This study is one of the three studies sanctioned by the Kenyan MOH in 2013 to generate relevant local evidence to inform decision-making on how iCCM can be implemented. Bondo Subcounty is in Siaya County in western Kenya. Bondo is an agrarian community with 26 Community Units and a population of about 250,000 inhabitants. Each Community Unit has about 5,000–10,000 inhabitants and is served by 1–2 CHVs depending on the population size and geographic spread of the villages based on the Kenya Community Strategy guidelines. Ethical approvals were obtained from institutional review boards at the Kenya Medical Research Institute and the Johns Hopkins Bloomberg School of Public Health, USA, before the commencement of the study.

### Study design

The iCCM main study employed a quasi-experimental design. Group assignment was based on pre-determined criteria of defining the hard-to-reach areas, ensuring that the intervention and comparison groups were at least 10 kilometres apart to minimize the risk of contamination. This manuscript utilizes a subset of the study data from the intervention group only: Fifty-eight CHVs in the four intervention group Community Units (East Migwena, West Migwena, Got Abiero, and Nyaguda). A pre-test/post-test design was used to assess changes in CHVs’ knowledge and skills in iCCM for the intervention group. The knowledge questionnaire and the observation checklist used to assess the CHVs was based on the standardized sick child health module commonly used in community knowledge, practice and coverage surveys [26]. Both tools were pretested before use.

### The intervention or care package

**Figure 2:**
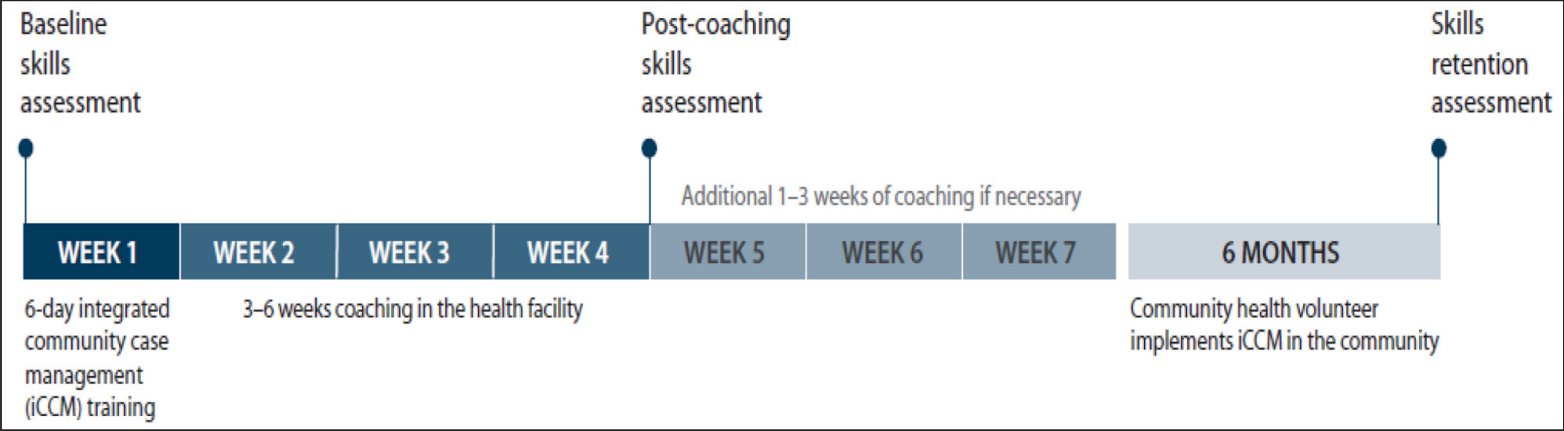
Intervention timeline.

The CHV capacity-building intervention comprised three components: a six-day skill-building training, clinical coaching at the health facility, and supportive supervision in the community. CHVs skills were assessed three times: at baseline, after at least three weeks of facility-based coaching, and after six months of implementing iCCM (see Figure 2).

#### Training

After pre-training skills and knowledge of childhood illness were assessed, all 58 CHVs were trained in iCCM for six days in a central location offsite by the Bondo Subcounty integrated Community Case Management trainers-of-trainers. Each day consisted of eight hours of learning divided into theory and practice. The training focused on imparting practical skills to a CHV to ask (for symptoms), look and assess (for signs), classify (assign illness) and treat (give home treatment or counsel caregiver and refer child) a child with symptoms of cough, diarrhoea, fever based on the iCCM algorithm which is an adaptation of the WHO Integrated Management of Childhood Illness (IMCI) algorithm (“an integrated approach to child health that focuses on the well-being of the whole child”) [27]. The training methods comprised lectures in a classroom setting on common childhood illnesses followed by hands-on practice sessions in a clinical setting through managing actual sick children and performing mRDTs under observation. In addition, participants engaged in role-play and case scenarios. The practicum took place at a non-study health facility. The trainers were Ministry of Health IMCI certified clinicians with skills/competencies in facilitation, performing mRDTs and adult learning techniques such as, eliciting feedback, role plays, approaches to ensuring active participation of all trainees. The trainer participant ratio was 1:6. The knowledge of CHVs was evaluated pre and post training while the skills were evaluated pre-training and after three weeks of clinical practice and coaching at the health facility.

#### Coaching

Clinical coaching followed immediately, starting the week after the six-day training. Each CHV spent three weeks managing sick children at a primary health facility under observation and being coached by either a nurse or a clinical officer trained in IMCI (“an integrated approach to child health that focuses on the well-being of the whole child” [28]) and oriented to the iCCM strategy. At the same time, laboratory technicians observed and coached the CHVs as they performed mRDTs.

The coaching took place in four health facilities that serve the population in the four Community Units’ catchment area included in the study. Two to three CHVs were assigned to each facility per day, Monday to Friday. The schedule depended on patient caseload and accommodated the facility staff as they continued performing their routine duties. This translated to each CHV spending 2 to 3 days per week at the assigned facility.

Coaching included guided practice in: **asking** about symptoms (cough, diarrhoea, and fever); **looking** and testing for signs of illness (breaths per minute [fast breathing], chest indrawing, lethargy, swelling of feet, mid-upper arm circumference); and deciding whether to treat or refer. The coach demonstrated relevant clinical techniques and signs to the CHV, who then performed the same duties under supervision. To ensure that CHVs were practicing all critical skills, each volunteer practiced on about three cases presenting with different complaints per day. Caregivers of sick children gave verbal consent to be part of the coaching sessions.

After three weeks of coaching, each CHV was assessed by a subcounty iCCM Trainer of Trainers on three to five iCCM cases. The overall coaching process ranged from 3–6 weeks depending on the number of iCCM-trained CHVs attached to the CU, number of coaches in a health facility, patient caseload, and CHV performance. All 58 CHVs were commissioned to provide iCCM services in the community when they met the requirements at different times between December 2013 and January 2014.

#### Supervision

The last component of the intervention was supportive supervision in the community for a period of 6 months, provided by community health extension workers (CHEWs). During this period, CHVs managed children presenting to them with symptoms of illness covered under iCCM and referred some to the health facility assigned to serve the CU catchment population (the link facility). Each month, the CHVs met with a CHEW for a coaching session at the link health facility. Together, they reviewed the data for the iCCM cases the CHVs had managed that month and the CHEW mentored the CHVs on identified gaps Each CHEW compiled a summary of all iCCM cases managed by the CHV in the monthly summary form.

In addition, CHEWs completed a mentorship form that documented the content of each meeting with each CHV. These data were used to identify the few community health volunteer who were not performing satisfactorily, and thus required further training back at the health facility where they had received additional coaching by health facility staff and released back to the community.

## Data collection

Data were collected in line with the intervention’s stages.

### Stage 1. Baseline skills assessment and pre- and post-training knowledge tests

Before the six-day training took place in September 2013, each CHV was observed while managing two clinical cases of children with iCCM conditions (fever, diarrhoea, cough/difficulty breathing). The trainers conducted the clinical assessment. For each case, the assessor used direct clinical observation, comparing the CHV performance to a locally developed standard CHV observation checklist, then re-examined the child.

On the first day of the six-day training, a pre-test was administered to assess the CHVs’ baseline knowledge in iCCM. A post-test was administered at the end of the six-day training. Each test had 25 questions. For each correct answer, the CHVs received one point; the total score for each CHV was then converted into a percentage. Skills were not reassessed at the end of the six-day training.

### Stage 2. Skills assessment following facility-based clinical practice and coaching

The CHVs’ clinical skills were again assessed by the Trainers in the health facilities after the CHVs had three weeks of coaching and practice in iCCM and mRDTs. The overall pass mark for clinical and mRDT skills assessed was 80%. CHVs who did not pass after the initial 3-week coaching period in December 2013 continued practicing the specific skills where they scored too low for an additional 1–3 weeks, until the health facility staff and CHEWs reassessed them in January 2014 and determined that they were competent to provide iCCM independently in the community.

### Stage 3. Skills retention assessment after 6 months of iCCM implementation

After six months of implementing iCCM, the CHVs were assessed by their trainers. This was done in the link health facilities. The Trainers followed the same skills assessment procedure applied at baseline: direct clinical observation of the CHVs managing a sick child and recording on a standard checklist and re-examination of the child.

## Data management and analysis

The CHV pre-test and post-test knowledge scores along with health facility coaching data were captured in Microsoft Excel spreadsheets. The baseline and six-month assessment data were captured on paper then entered into an electronic database—the Census and Survey Processing System [29]. Data were cleaned, coded (where necessary), and then exported to STATA®for analysis [30,31]. For each of the knowledge tests, each correct answer earned the CHV one point (out of 25); the total score was then converted into a percentage. Changes in knowledge test scores from pre-test to post-test were computed.

Frequency tables for the demographic characteristics, box plots, and charts were generated to summarize the data. To test improvements in CHV knowledge before and after the six-day training, paired t-test comparison of means was done; the level of significance was set at 5%. A test of proportions was used to define any significant improvement in the number of CHVs able to carry out proper diagnosis before and after receiving training. Data were documented in an Excel spreadsheet and analysed using Stata.

Key variables of interest during data analysis were broadly categorized into CHV knowledge and skills in: 1) asking about symptoms of illnesses, 2) assessing/identifying/classifying signs and symptoms of childhood illnesses, 3) correctly measuring mid-upper arm circumference and performing mRDTs, 4) making the decision to treat or refer, and 5) correctly treating diarrhoea or fever.

## Results

### Baseline Characteristics of CHVs

All 58 CHVs participated in baseline clinical skills assessment, pre-test/post-test iCCM knowledge assessment, and clinical coaching at the health facility. The majority were female (82.8%), aged 20–50 years (77.6%), and had completed primary school (about 8 years of education; 81.0%). The baseline characteristics of the CHVs are summarized in Table 1.

**Table 1:** Characteristics of the community health volunteers (CHVs; N = 58).

	Number	Percentage

**Gender**		
Female	48	82.8
Male	10	17.2
**Education level**		
Primary school	47	81.0
High school	11	19.0
**Age in years**		
20–35	18	31.0
36–50	27	46.6
51–65	12	20.7
65+	1	1.7
**Years as Community Health Volunteer**		
<1	1	1.7
1–7	36	62.1
8–14	10	17.2
15–21	6	10.3
21+	5	8.6
**Previous training (multiple responses were allowed)**		
Family planning	48	82.8
HIV/prevention of mother-to-child transmission	12	20.7
Infant and young child feeding	12	20.7
Malaria case management/testing	5	8.6
Multidrug-resistant TB	5	8.6
Other training beyond the basic package: * home-based care, malaria prevention, neonatal case management, prevention for positive, ^†^ post abortion care, palliative care.	40	69.0

* Ministry of Health. Taking the Kenya Essential Package for Health to the community: a strategy for the delivery of level one services. Ministry of Health, Health Sector Reform Secretariat 2006. http://www.communityledtotalsanitation.org/sites/communityledtotalsanitation.org/files/community_strategy.pdf Accessed 28 Feb 2017. ^†^ “Prevention for positive” refers to equipping HIV-positive individuals with knowledge/information to live a lifestyle that prevents them from re-infection.

In addition, nearly two-thirds of them had volunteered in the community for 7 years (interquartile range: 3–10 years).

### Pre and Post Assessment Results

**Figure 3:**
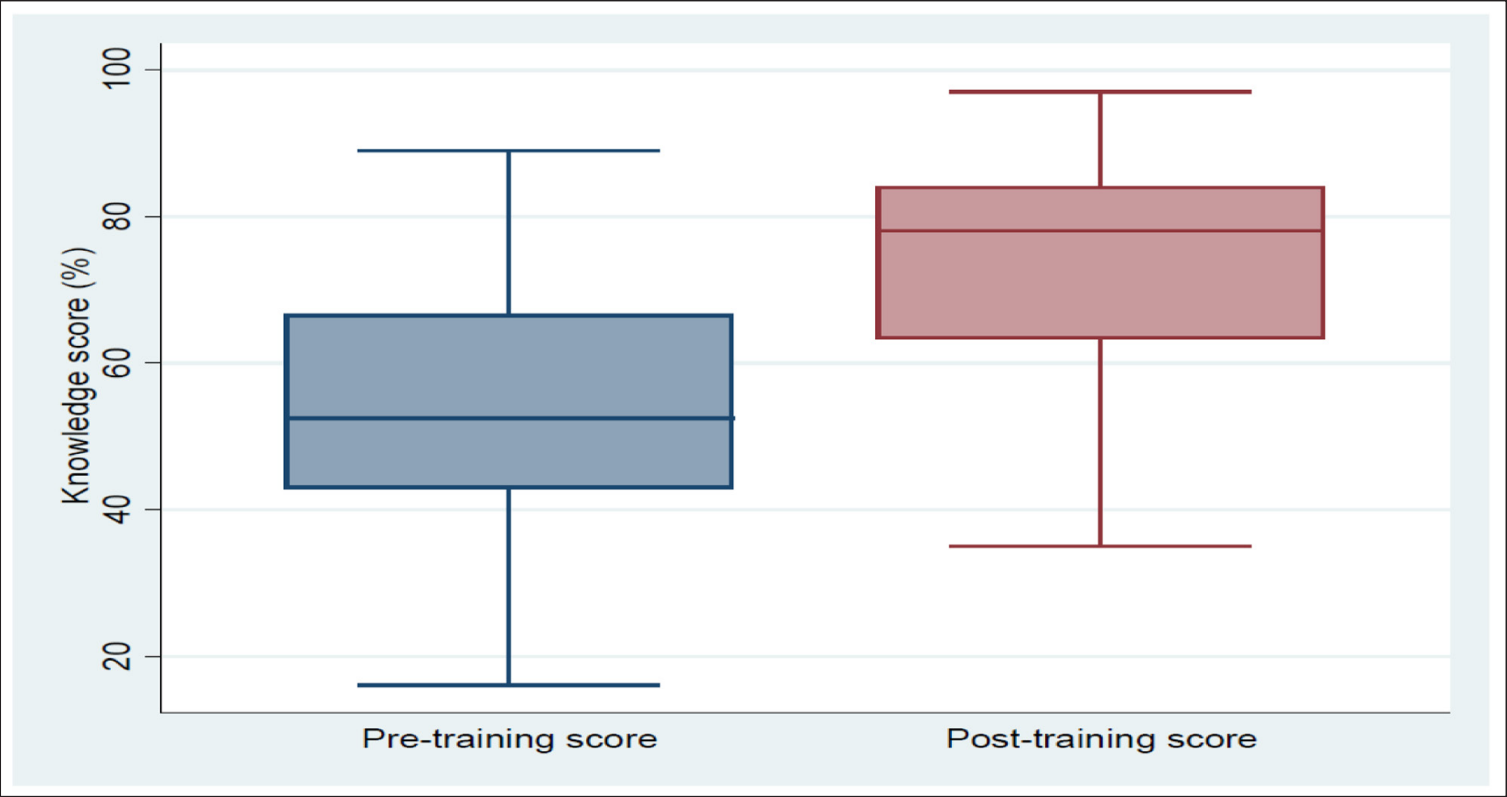
Effect of six-day training in iCCM on community health workers’ knowledge.

All 58 CHVs were assessed on knowledge of signs, symptoms, and treatment of suspected pneumonia, diarrhoea, and malaria, including identification of danger signs. The pre-test had 25 questions and for every response that a CHVs answered correctly, a score of one was assigned. The total score for each CHVs was summed and converted into a percentage. There was a significant improvement in knowledge scores after the six-day training from a pre-training median score of 54.5% (interquartile range 44.5%–64.4%) to a post-training median score of 72.9% (interquartile range 61.5%–84.3%), representing an overall 18 percentage point increase (*p*-value < 0.001) as shown in Figure 3.

### CHVs Performance Assessment at 3–6 weeks

After 3–6 weeks of clinical coaching at the link health facilities, all 58 CHVs could assess and classify fever and diarrhoea correctly, 97% could identify malnutrition correctly, and 80% could assess and classify suspected pneumonia (with fast breathing used as a proxy measure) correctly. Proper and timely diagnosis is essential to the management of childhood illnesses, especially fever.

**Figure 4:**
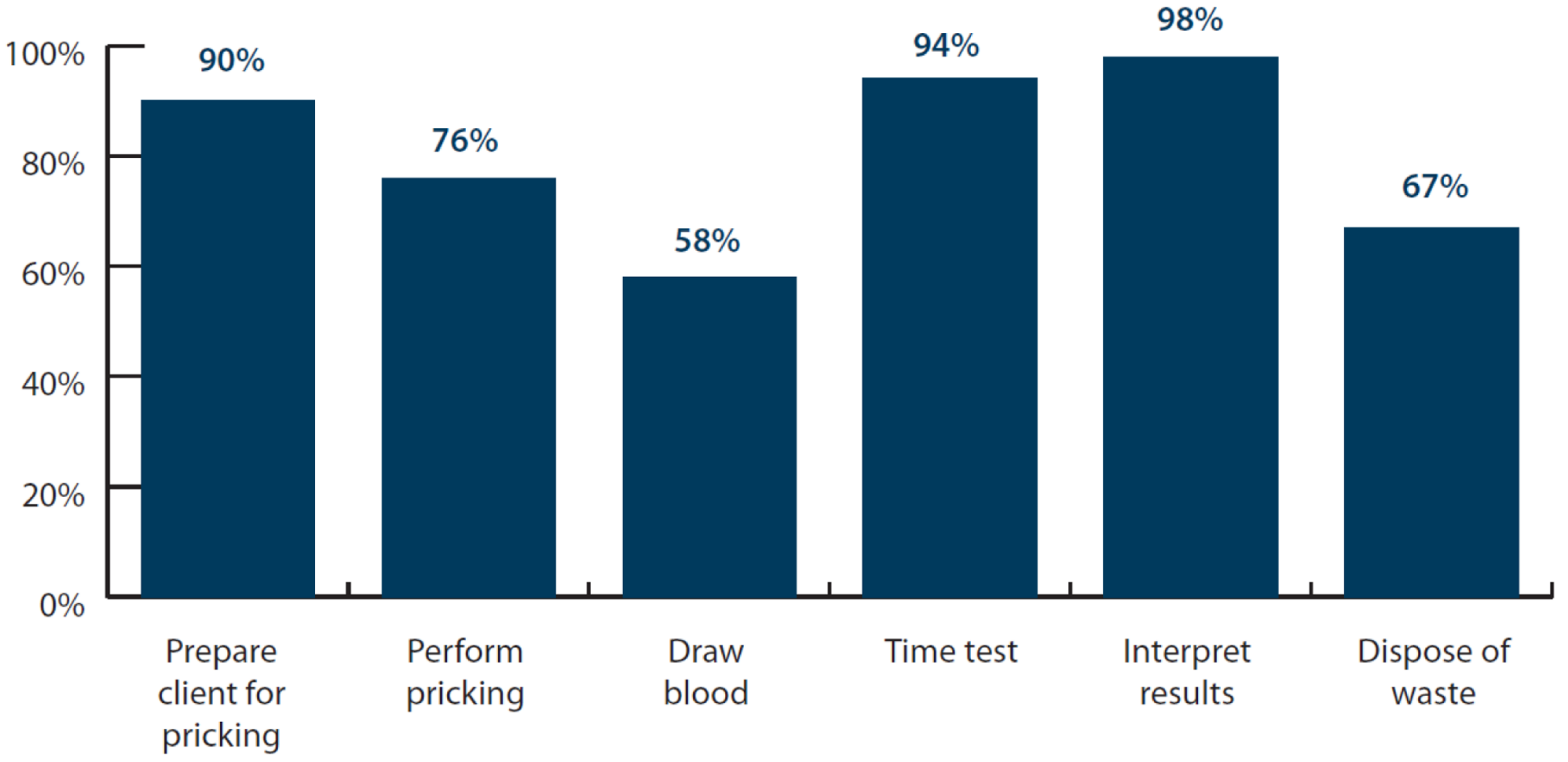
Percentage of CHVs correctly performing steps of mRDT.

Each CHV was observed and assessed performing a mRDT on three occasions during the coaching period, on average, to determine competency. Most CHVs could perform at a high level on four of the six mRDT steps, which should be done in the proper sequence. However, only 58% could draw blood correctly and 67% correctly dispose of waste after performing the test (Figure 4).

There was a low score of 58% success in drawing blood while correct performance of all other steps was above 60 percent.

### CHV Performance Assessment at Six Months

After six months of implementing iCCM, three CHVs dropped out for various reasons; thus, 55 CHVs were assessed on iCCM clinical skills at 6 months. The CHVs demonstrated ability to follow correctly the Kenya iCCM algorithm [21] from identifying signs through classifying the illness and deciding whether to treat at home or refer to the health facility. The greatest improvement was in the ability of CHVs to examine or look for signs of illness (average of 4% at baseline vs. 74% at 6 months, *p* < 0.05).

Regarding the decision to treat or refer after identification of danger sign(s), there was a marked increase in assessors’ agreement with the CHVs’ decisions, from 13% at baseline to 67% at 6 months. Out of the nine children recommended for referral at the 6-month assessments, the assessor agreed with the CHVs decision to refer in six instances. The CHVs’ ability to recognize when danger signs were not present—as validated by the assessor’s agreement—also improved, from 77% at baseline to 98% at 6 months (*p* < 0.05). At 6 months, the assessor agreed with the CHVs decision to refer 82 out of 84 times. The changes in the CHVs’ clinical skills from baseline to six-month assessments are shown in Table 2.

**Table 2:** CHV iCCM skills at baseline and after 6 months.

Category of assessment	Baseline observations (N = 115)*		6-month observations (N = 106)^†^		P-value^‡^

	Yes	%	Yes	%	

**CHV asked about symptoms**					
Cough	102	89%	98	92%	0.341
Diarrhoea	86	75%	89	84%	0.930
Fever	110	96%	103	97%	0.547
**Average**		87%		91%	0.344
**CHV looked for signs of illness**					
Chest indrawing	3	3%	75	71%	**0.001**
Fast breathing by counting breaths in 1 minute	0	0%	61	58%	**0.001**
Unusually sleepy or lethargic or unconscious child	4	3%	72	68%	**0.001**
Malnutrition using the mid-upper arm circumference tape colour code	11	10%	95	90%	**0.001**
Malnutrition using the thumbs to press and demonstrate swelling of both feet	2	2%	88	83%	**0.001**
**Average**		4%		74%	**0.001**
**Classifying danger signs and deciding to treat or refer**					
CHV classified child as having any danger sign and decided for urgent referral	15		9		
Assessor agreed with CHV classification of danger sign and decision to refer	2/15	13%	6/9	67%	**0.001**
CHV classified child as having no danger sign and decided for home treatment and advice to caregiver	13		84		
Assessor agreed with CHV classification of no danger sign and decision for home treatment and advice to caregiver	10/13	77%	82/84	98%	**0.001**

* 58 CHVs were assessed for a total of 115 observations; average of two observations per CHV. ^†^ Three CHVs had dropped out, so 55 were assessed for a total of 106 observations; average of two observations per CHV. ^‡^ Two-sample test of proportions *p*-value.

Fever was classified and managed based on duration and mRDT results. Additional monitoring data collected during the first six months of study showed that most of the fever cases presented were <7 days’ duration (1,544/1,549; 99.7%). CHVs performed the mRDTs and treated the children with a positive result, the majority at 1,524/1,544 (98.7%). In addition, the CHVs examined 538 children with diarrhoea, all without blood in their stools and with less than 14 days of the symptom. Ninety-three percent of these cases were managed at home with ORS and zinc.

## Discussion

This study describes an approach used to build CHVs’ competence to provide iCCM as a task-shifting strategy to mitigate chronic shortages of professional health workers, especially in the underserved areas of Kenya. While it is relatively easy to teach lay health workers health promotion and appropriate preventive practices, providing case management is what distinguishes professional health workers from lay people. Acquiring the clinical skills to effectively manage sick children takes months of supervised practice in the health facility and implementation in the community [16]. The shortage of professional health workers also reduces the ability to supervise trained CHVs when they are providing iCCM. Therefore, an approach that ensures CHV mastery of skills before independent practice assures that quality care is provided.

The key finding of this study is that a CHV skills-building approach that combines initial training, facility-based practice and coaching before the CHVs are commissioned to provide iCCM independently, and continuous supportive supervision during implementation, improves skills mastery and retention. The assessment of skills at baseline and six months used direct clinical observation, thus providing a high level of confidence that the CHVs acquired the skills. In this study, we assumed that the initial training, for six days without assurance of regular supervision, is inadequate to build clinical skills for CHVs to practice independently. This study therefore used a two-stage approach that added coaching of CHVs at the health facility by clinical staff and supportive supervision for 6 months. Overall, the results show this approach as more promising than training alone to building clinical skills of CHVs especially when regular supervision that includes case management observation is not assured. However, further studies that compare single-step and multi-step approaches to training are needed to guide program managers on cost-effective approaches.

This study adds to existing practical knowledge and experiences in building skills of lay health workers, including identifying symptoms and signs of illness, performing the mRDT, and deciding to provide home treatment or to refer to a higher-level facility.

### Profile of Community Health Volunteers and integrated Community Case Management knowledge

In this study, most CHVs had primary level general education and were in their economically productive years (ages 20–50), both of which factors could have helped their ability to grasp the concepts and skills. The pre-training knowledge test scores before iCCM training were relatively high, above 50%, and increased to over 70% after the six-day training. Previous training in a variety of health packages such as infant and young child feeding, malaria prevention, and neonatal case management may have contributed to the relatively high levels of pre-training knowledge noted. In addition, about a tenth of the CHVs had been trained in malaria case management prior to this training. In some settings, CHVs are illiterate or have low literacy, which poses a challenge when training them to carry out the clinical tasks required for iCCM. Less literate CHVs may be slow in comprehending the subject matter. In such cases, use of pictures and symbols to demonstrate clinical signs during training and supervision enhances acquisition of knowledge and skills of childhood illness (26). The study did not explore CHVs self-confidence.

### Acquisition of clinical skills

To prevent death, CHVs should recognize signs of illness and danger signs and recommend appropriate timely care. After six months, all fevers were correctly identified and in more than two-thirds of cases assessed, children who were unusually sleepy or unconscious (a danger sign) were recognized. The high level of skills in fever identification in our study compares well with the 97% reported in the Mukanga et al. study in Uganda [16]. A critical sign of pneumonia, a leading cause of child deaths in Sub-Sahara Africa and South East Asia is chest indrawing (27). No CHV looked for this sign at baseline. After 6 months, CHVs looked for chest indrawing in over 70% of cases observed. Counting breaths per minute and comparing to a standard for the child’s age is diagnostic of pneumonia when the breaths per minute exceed a set standard for different age bands. In this study, CHVs correctly counted breaths in just a little over half of cases when assessed at six months. This score is low compared to other skills.

Standard design of iCCM assumes that CHVs will be supervised, including case observation assessments and mentoring while providing services. However, in practice, scheduled supervision and mentoring don’t take place as planned due to limited resources and tasks competing for the time of the supervisors. Investing the extra time to enforce skills through facility-based coaching is a promising approach that could compensate for the lack of regular, personalized supportive supervision and mentoring once CHVs are providing case management in the community. In addition, due to the limited resources, when at scale, most iCCM programs end up providing group rather than individualized supervision. In such cases, the opportunity to focus on CHVs’ individual weaknesses is lost.

The skills-building model used in this study is however, both labour- and time-intensive. It required effort and goodwill from health facility staff as well as CHVs. Notwithstanding, the coaching relationship allows the CHV to master skills and confidence, which is unlikely during the six-day training. The training approach assumes all CHVs to be at the same level before the training; individualized skills building is not possible because the trainers must deliver each day’s curriculum on schedule. Coaching not only builds a relationship between facility staff and the CHVs, but also ensures direct involvement of facility-based staff in community health activities. As the CHVs improved their skills, they were performing tasks to complement facility staff. The advantage is that this approach assures a strong foundation for CHVs to ensure that they can function with limited support which is the reality of iCCM (and other health services) at scale. The feasibility and acceptability of the model to the health system can only be assured after it has been used to build the skills of many CHVs in a government-led program with limited or no project support. This coaching model can also be adapted to follow group-based refresher training: CHVs identified to need further coaching can be assigned a coach at the health facility. This can be an ongoing process based on the need of the individual CHVs.

Among the limitations of the study is that the testing of this approach did not have a comparison group (six-day training only) and did not include evaluation of clinical skills after the six days training. However, the findings support the hypothesis that training, facility-based coaching, and regular supportive supervision contributed to the high skills acquired and maintained at six months. Another limitation is that we did not specifically quantify the direct and indirect costs of coaching, including any negative effects of the disruption to services, change in clients’ waiting time and caregivers’ feedback on experience of care, etc. during the facility-based coaching period. We also did not get structured feedback from the coaches/mentors on the approach and both its positive and negative impacts on services. However, the findings provide valuable insights on the implementation of a capacity building of non-clinical staff to carry out clinical procedures through task-shifting to increase access to life-saving interventions.

## Conclusions

Overall, the CHVs acquired iCCM skills and demonstrated competency in providing iCCM after training, facility-based coaching, and supportive supervision. CHVs demonstrated the ability to correctly follow the iCCM algorithm [22] and decide whether to treat at home or refer to the health facility. We recommend further testing and documentation of this coaching approach as it has potential to inform implementation and scale-up of iCCM. We also recommend a cost benefit analysis of this coaching approach.
